# Modeling time-to-cure from severe acute malnutrition: application of various parametric frailty models

**DOI:** 10.1186/2049-3258-73-6

**Published:** 2015-01-01

**Authors:** Akalu Banbeta, Dinberu Seyoum, Tefera Belachew, Belay Birlie, Yehenew Getachew

**Affiliations:** Department of Statistics, College of Natural Science, Jimma University, Jimma, Ethiopia; Department of Population and Family Health, College of Public Health and Medical Science, Jimma University, Jimma, Ethiopia

**Keywords:** Severe acute malnutrition, Parametric frailty, Accelerated failure time model

## Abstract

**Background:**

In developing countries about 3.5% of children aged 0–5 years are victims of severe acute malnutrition (SAM). Once the morbidity has developed the cure process takes variable period depending on various factors. Knowledge of time-to-cure from SAM will enable health care providers to plan resources and monitor the progress of cases with SAM. The current analysis presents modeling time-to-cure from SAM starting from the day of diagnosis in Wolisso St. Luke Catholic hospital, southwest Ethiopia.

**Methods:**

With the aim of coming up with appropriate survival (time-to-event) model that describes the SAM dataset, various parametric clustered time-to-event (frailty) models were compared. Frailty model, which is an extension of the proportional hazards Cox survival model, was used to analyze time-to-cure from SAM. Kebeles (villages) of the children were considered as the clustering variable in all the models. We used exponential, weibull and log-logistic as baseline hazard functions and the gamma as well as inverse Gaussian for the frailty distributions and then based on AIC criteria, all models were compared for their performance.

**Results:**

The median time-to-cure from SAM cases was 14 days with the maximum of 63 days of which about 83% were cured. The log-logistic model with inverse Gaussian frailty has the minimum AIC value among the models compared. The clustering effect was significant in modeling time-to-cure from SAM. The results showed that *age* of a child and *co-infection* were the determinant prognostic factors for SAM, but *sex* of the child and the *type of malnutrition* were not significant.

**Conclusions:**

The log-logistic with inverse Gaussian frailty model described the SAM dataset better than other distributions used in this study. There is heterogeneity between the kebeles in the time-to-cure from SAM, indicating that one needs to account for this clustering variable using appropriate clustered time-to-event frailty models.

## Background

Malnutrition is a common cause of morbidity and mortality in developing countries [1]. It is a risk factor for over 50% of the 11 million annual childhood deaths [2]. Severe acute malnutrition (SAM) is defined as a very low weight for height less than 70% NCHS median or less than 115 mm of MUAC [3]. In developing countries, about 3.5% of children aged 0–5 years are suffer from SAM [4], which is the most important nutritional disease because of its high prevalence and relationship with child mortality [5]. Currently, there are a number of studies on time of recovery from acute malnutrition in various parts of the world. Survival data is a term used for describing data that measure the time to a given event of interest. The term *survival data* is often used for describing data that measure the time to the occurrence of a given event of interest. The event of interest can be seen as a transition from one state to another. In this study, the event of interest was the time-to-cure from SAM from the day of diagnosis. One of the major aim of this analysis was to model the time-to-cure from SAM among less than five year inpatient children in Wolisso St. Luke Catholic hospital and to compare the efficiency of various parametric frailty models using the same dataset. The classical model for this kind of data is the proportional hazards model popularized by Cox [6]. However, correct inference based on Cox’s model needs identically and independently distributed samples. One of the reasons why this model is so popular is the ease with which technical difficulties such as censoring and truncation are handled. This is due to the appealing interpretation of the hazard as a risk that changes over time. Naturally, the concept allows for entering covariates to describe their influence and model different levels of risk for different subgroups. Nonetheless, subjects may be exposed to different risk levels, even after controlling for known risk factors; as some relevant covariates were often unavailable to the researcher or even unknown (univariate frailty case). The study population may also be divided into clusters so that subjects from the same cluster behave more cohesively than subjects from different clusters (multivariate frailty case). The frailty model, introduced in the statistical literature by Vaupel et al. [7], and discussed in details [8–10], accounts for heterogeneity in baseline. It is an extension of the proportional hazards of Cox’s model in which the hazard function depends upon an unobservable random quantity, the so-called frailty that acts multiplicatively on it. Study subjects (children) in this study came from clustered community and hence clustered child survival data may be correlated at the Kebele level. In this research, shared frailty models were explored assuming that children within the same cluster (kebele) share similar risk factors, which will take care of the frailty term at kebele level. This model is a conditional independence model where the frailty is common to all individuals in a cluster, and therefore responsible for creating dependence between event times. This is because ignoring the full dependence among observations may lead to standard errors that are understated and parameter estimates that are both biased and inconsistent [11]. Estimation of the frailty model can be parametric or semi-parametric. In the first case, a parametric density is assumed for the event times, resulting in a parametric baseline hazard function. Estimation is then conducted by maximizing the marginal log-likelihood function. In the second case, the baseline hazard is left unspecified and more complex techniques are available to approach that situation [12]. Even though semi-parametric estimation offers more flexibility, the parametric estimation will be more powerful if the form of the baseline hazard is somehow known in advance [13]. In this study, parametric frailty models were used to investigate the relationship between different potential covariates (*sex*, *age*, *type of malnutrition* and *co-infection*) and time to cure from SAM for clustered survival data with random right censoring. The choice of distribution for the hazard is very important than the choice of frailty distribution [14]. Hence, in this research exponential, weibull and log-logistic hazard functions were used and compared for their efficiencies. Regarding the frailty distribution, we assumed gamma and inverse Gaussian distributions. For comparison of different distributions, the AIC criteria were used, but for comparing nested models, likelihood ratio test were used.

## Methods

### Study sample and setting

The data set used in this study were obtained from Wolisso St. Luke catholic hospital, Wolisso, south west Ethiopia. Children aged under five, having marasmus and/or kwashiorkor, diagnosed for severe acute malnutrition (SAM) according to the protocol for management of SAM [15] and admitted to inpatient, and started treatment during the period January 1, 2010 to January 31, 2012 were eligible for the analysis. A total of 929 children who came from 280 kebeles around Wolisso district were considered. Kebeles that contribute only a child were omitted since the shared frailty model should be done on at least two children per kebele. Therefore a total of 855 children with severe acute malnutrition from 206 kebeles were considered.

The response variable time-to-cure from SAM were obtained by calculating the difference (in day) from the start of treatment until the child were cured (recovered) or censored. Cured children according to the SAM treatment protocol are defined as children who have weight for height >85% and no bilateral edema. Children were considered to be cured and discharged, which is our event of interest, if they fulfilled the discharging criteria for SAM [15]. However, the time-to-cure were censored for those children transferred to other hospital, dropped treatment, died, did not cure at January 31, 2012 (at the end of study). The following variables were considered for their influence on the time-to-cure from SAM; *sex*, *age*, *type of malnutrition* and *co-infection*. For *age* we used six categories; 0–5 months, 6–11 months, 12–23 months, 24–35 months, 36–47 months and 48–59 months. *Types of malnutrition* were categorised as Marasmus, Kwashiorkor and Marasmic-kwashiorkor. *Co-infection* was categorized based on whether the child has co-infection such as malaria, anemia, pneumonia, measles and giardiasis or not.

### Shared frailty model

Conditional on the random term, called the frailty denoted by *u*_*i*_, the survival time in cluster *i* (1≤*i*≤*n*) are assumed to be independent, the proportional hazard model assumes


Whereas an alternative if the proportional hazards assumption does not hold is the accelerated failure time frailty model which assumes


Where *i* indicates the *i*^*th*^ cluster, *j* indicates the *j*^*th*^ individual in the *i*^*th*^ cluster, *h*_0_(*t*) is the baseline hazard, *u*_*i*_ the random term for all subjects in cluster *i*, *X*_*ij*_ is the vector of covariates for subject *j* in cluster *i*, and ***β*** the vector of regression coefficients.

We assumed that *Z* (where *Z* = *exp*(*u*_*i*_)) has the gamma or inverse Gaussian distribution so that the hazard function depends upon this frailty that acts multiplicatively on it. The main assumption of a shared frailty model is that all individuals in cluster *i* share the same value of frailty *Z*_*i*_(*i* = 1,…,*n*), and that is why the model is called the shared frailty model. The survival time is assumed to be conditionally independent with respect to the shared (common survival times) frailty. This shared frailty is the cause of dependence between survival time within the clusters.

In order to investigate the effect of the candidate covariates on the time-to-cure from SAM, we first did a univariable analysis by fitting a separate model for each candidate covariates. Covariates that were found to be significant in the univariable analysis were included in the multivariable analysis.

The multivariable survival analysis in the study was done by assuming the exponential, weibull and log-logistic distributions for the baseline hazard function; and the gamma and inverse Gaussian frailty distributions. It was performed using the three most significant covariates in the univariable analysis namely *age*, *type of malnutrition* and *co-infection*. However, we excluded *sex* which was not significant in univariable analysis.

## Results

Of all 855 malnourished patients, 711(83.16%) were cured and the median cure time from SAM was 14 days, while the minimum and the maximum cure times observed were 7 and 63 days, respectively (Table 1).

**Table 1:** **Descriptive summaries of patient’s characteristics diagnosed for SAM**

Characteristic		No. of patients	Cured (%)	Median (days)	(95% CI)
Sex	Female	421	348(82.66)	15	(14,16)
	Male	434	363(83.64)	14	(13,15)
Age group	0–5 months	61	46(75.4)	13	(12,17)
	6–11 months	196	152(77.5)	15	(13,17)
	12–23 months	281	242(86.1)	16	(15,17)
	24–35 months	152	128(84.2)	14	(13,17)
	36–47 months	82	77(93.9)	13	(11,15)
	48–49 months	83	66(79.5)	13	(11,16)
Type of Malnutrition	Marasmus	399	314(78.7)	15	(14,17)
	Kwashiorkor	382	342(89.53)	14	(13,15)
	Marasmic-kwashiorkor	74	55(74.3)	15	(14,20)
Co-infection	No	376	335(89.1)	13	(12,14)
	Yes	479	376(78.5)	17	(16,18)
Total		855	711(83.16)	14	(14,15)

Using all the multivariable frailty models, the covariate *co-infection* was significant, indicating that it was the most important prognostic factor for the time-to-cure from SAM. *Age group* was significant in the three models namely, weibull-gamma, weibull-inverse Gaussian and log-logistic-inverse Gaussian frailty models. *Type of malnutrition* was not a significant factor for time-to-cure from SAM using all the models. The variance of the random effect (*θ*) was significant for the weibull and log-logistic baseline frailty models but not significant for the exponential baseline frailty models. It was highest when we assume the inverse Gaussian distribution (*θ* = 0.21) followed by the gamma distribution (*θ* = 0.172) with the log-logistic baseline hazard function. This term was again higher when we assume the inverse Gaussian frailty distribution (*θ* = 0.169) than the gamma distribution (*θ* = 0.163) for the weibull baseline hazard function. The Kendall’s tau (*τ*) was higher for the higher *θ* values. Accordingly the dependence within the clusters for the log-logistic-inverse Gaussian frailty model (*τ* = 0.081) was the maximum followed by the log-logistic-gamma frailty model (*τ* = 0.079). The AIC value of the log-logistic-inverse Gaussian model 4941.63, was the minimum among all the other AIC values of the models indicating that it was the most efficient model to describe the SAM dataset using various parametric frailty models (Table 2).

**Table 2:** **AIC values of the parametric frailty models**

Baseline hazard function	Frailty distribution	AIC
Exponential	Gamma	5534.547
	Inverse-Gaussian	5535.706
Weibull	Gamma	5052.727
	Inverse-Gaussian	5014.042
Log-logistic	Gamma	4997.691
	Inverse-Gaussian	4941.630

Analysis based on log-logistic-inverse Gaussian frailty model showed that *age group* of the children and presence of *co-infection* were significant. That is the confidence interval of acceleration factors(*ϕ*) for both *Age group* and *co-infection* did not include 1 (Table 3).

**Table 3:** **Multivariable analysis using the log-logistic-inverse Gaussian frailty model**

Covariates		Coefficients	S.E.	***ϕ***	95 ***%*** CI of ***ϕ***
Intercept		2.542	0.072	12.711	(11.030, 14.648)*
Age group	0–5 months	Ref		1.000	
	6–11 months	0.036	0.079	1.037	(0.888, 1.210)
	12–23 months	0.166	0.078	1.180	(1.014, 1.374)*
	34–35 months	0.064	0.085	1.066	(0.903, 1.258)
	36–47 months	-0.022	0.092	0.979	(0.817, 1.173)
	48–59 months	0.028	0.094	1.029	(0.856, 1.236)
Type of Malnutrition	Marasmus	Ref		1.000	
	Kwashiorkor	-0.045	0.044	0.956	(0.876, 1.043)
	Marasmic-kwashiorkor	0.014	0.070	1.014	(0.884, 1.165)
Co-infection	No	Ref		1.000	
	Yes	0.163	0.038	1.177	(1.093, 1.268)*
	log(scale) = -1.268 (0.031)*				*τ* = 0.081
	*θ* = 0.21(*S*.*E* = 0.055)∗				*λ* = 1.306*e* ^-4^
	*ρ* = 3.56(*S*.*E*. = 0.134)				*AIC* = 4941.630

Source: Wolisso St. Luke Catholic hospital, Ethiopia; from September 1, 2010 to January 31, 2012. *p < 0.05 was statistically significant. *ϕ* = Acceleration factor, *θ* = Variance of the random effect, *τ* = Kendall’s tau, AIC = Akaike’s Information Criteria, CI = confidence interval, S.E = standard error, Ref = Reference, *λ* = scale, *ρ* = shape.

Children with age group between 12 and 23 months had significantly different curing time than the reference groups (aged 0–5months) with acceleration factor (*ϕ* = 1.18) and confidence interval (1.014, 1.374), did not includes 1, at 5% level of significance. An acceleration factor of greater than 1 indicates prolonging the time-to-cure from SAM. Therefore, children aged between 12 and 23 months had prolonged cure time by a factor of 1.18 than the reference groups. But the other age groups were not significantly different from the baseline age group at 5% level of significance. The confidence interval of the acceleration factor of *co-infection* was (1.093, 1.268), did not include 1, indicating *co-infection* is also significant prognostic factor for time-to-cure from SAM. It prolonged curing time by a factor of (*ϕ* = 1.177) at 5% level of significance.

The value of the shape parameter in the log-logistic-inverse Gaussian frailty model was (*ρ* = 3.56). This value greater than unity indicates that the shape of hazard function is unimodal, i.e., it increases up to some time and then decreases. The variability (heterogeneity) in the population of clusters (kebeles) estimated by our working model was *θ* = 0.21, and the dependence within clusters was about *τ* = 8.1*%*.

**Figure 1:**
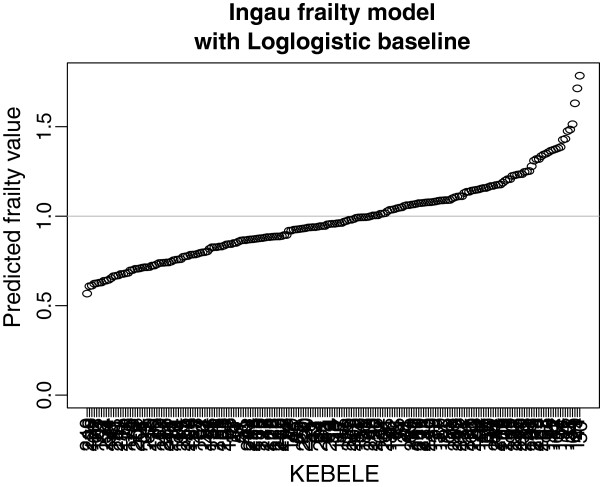
**Prediction of frailties for the SAM dataset as given by the parametric log-logistic-inverse Gaussian frailty model.**

The predicted frailty values increases with range of 0.731 to 1.272 with increasing the median time of the cluster on log-logistic- inverse Gaussian frailty model (Figure 1). That is, these values are lower for lower values of event times and higher for higher value of event times. The median value of the frailty distribution is around 1.

**Figure 2:**
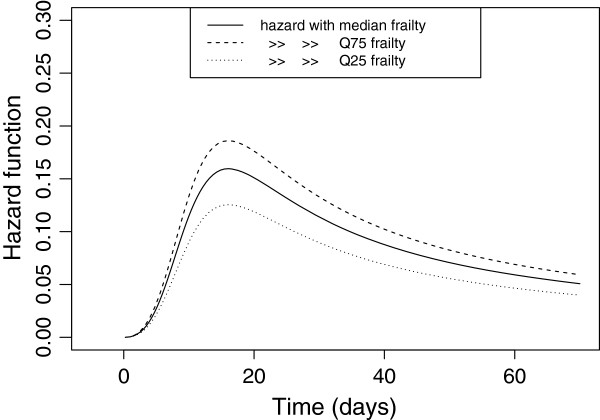
**Conditional hazard rates of the log-logistic- inverse Gaussian frailty model for the SAM dataset.**

The hazard functions given the 25^*th*^, 50^*th*^ and 75^*th*^ quartiles of frailty distribution (*Z* = 0.837,*Z* = 1*andZ* = 1.167, respectively) were plotted for the log-logistic-inverse Gaussian frailty model (Figure 2). The parameter estimates obtained by maximizing the likelihood of the log logistic-inverse Gaussian frailty model were *λ* = 1.306*e*-4, *ρ* = 3.56(*S*.*E* = 0.134) and *θ* = 0.21(*S*.*E* = 0.055).

The conditional hazard functions given the 75^*th*^ quartile frailty values was greater, followed by the conditional hazard functions given by the 50^*th*^ (median) and 25^*th*^ quartile frailty values of the clusters respectively. From this what we can observe is that the more frail groups (*Z* > 1) were more likely to get events earlier (cure earlier) and the less frail groups (*Z* < 1) had prolonged cure time. All the conditional hazard functions were almost equal at the beginning time (*t* = 0). But the gap widens through time, specifically at mid time. The pattern of all the hazard function is unimodal (increases up to some point and then decreases) as the shape parameter for the baseline hazard function is greater than unity (*ρ* = 3.56).

**Figure 3:**
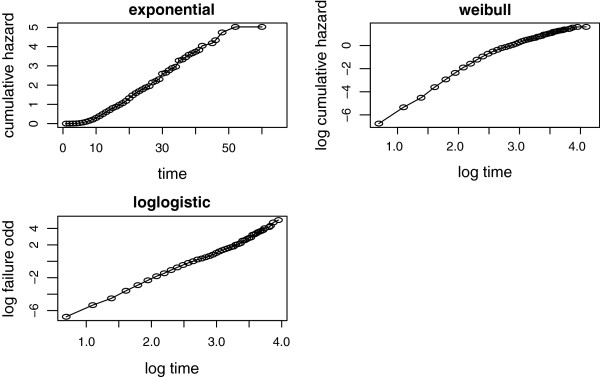
**Graphical evaluation of the exponential, weibull and log-logistic assumptions.**

To check the adequacy of our baseline hazard, the exponential has been plotted by the cumulative hazard function with time-to-cure from SAM. Similarly, the weibull has been plotted by the logarithm cumulative hazard function with the logarithm of time-to-cure from SAM and log-logistic has been plotted by the logarithm of the failure odds with the logarithm of time-to-cure from SAM (Figure 3). The plot of log-logistic was more linear than the other plots, though only few observations were scattered at the beginning time. The patterns suggested that the log-logistic hazard function was appropriate in the model.

**Figure 4:**
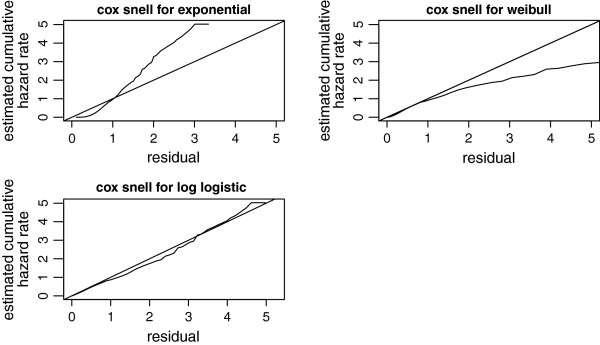
**Cox-Snell residuals obtained by fitting exponential, weibull and log-logistic models to the SAM dataset.**

The Cox-Snell residuals together with their cumulative hazard function were obtained by fitting the exponential, weibull and log-logistic models to our dataset, via maximum likelihood estimation (Figure 4). The plots showed that the Cox-Snell residuals fitted to assess the log-logistic model for the dataset were nearest to the line through the origin as compared to the other models, again indicating that this model described the SAM dataset well.

**Figure 5:**
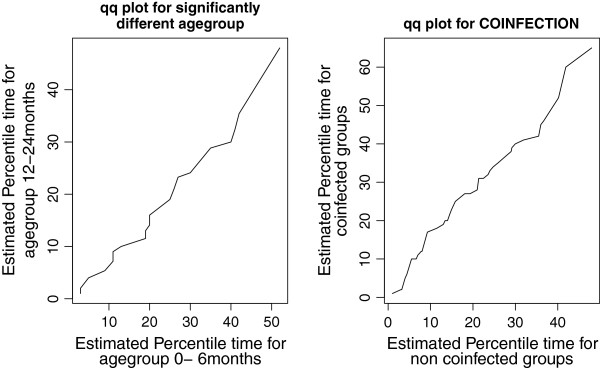
**q-q plot to check the adequacy of the accelerated failure time model.**

A quantile-quantile or q-q plot was made to check if the accelerated failure time provided an adequate fit to the data using two different groups of population. We checked the adequacy of the accelerated failure-time model by comparing the significantly different age groups (children in the age group 0–5 months and 12–24 months); as well as the co-infected and non co-infected groups of patients (Figure 5). The figures appear to be approximately linear for both covariates *age group* and *co-infection* with slopes equivalent to the acceleration factors 1.180 and 1.177, respectively. Therefore the log-logistic baseline model used for time-to-cure from SAM was accelerated failure time model.

## Discussion

The main aim of the study was to model time-to-cure from SAM using appropriate survival model among various parametric frailty models. The comparison of distributions of the models was performed using the AIC criteria, where a model with minimum AIC is accepted to be the best [13]. Accordingly, the log-logistic-inverse Gaussian frailty model which has AIC value of 4941.63 was the most appropriate model to describe the SAM dataset.

This study also showed that there was a clustering (frailty) effect on modeling time-to-cure from SAM which might be due to the heterogeneity in kebele from which the child came, assuming children coming from the same kebele share similar risk factors related to SAM. Therefore, it was important considering the clustering effect in modeling the hazard function. Clusters with minimum median time have smaller frailties, so that these clusters are predicted to have a high hazard [9], more probable to cure in this case. Our data demonstrated that the nuisance (frailty) terms modified the hazard function, and therefore the the hazard function was evaluated conditionally on this effect. Kebeles that frail more were more likely to cure than the less frail kebeles (since the event is positive).

The inverse Gaussian frailty generates very strong dependence at mid time [16]. According to the conditional hazard function given the 25^*th*^, 50^*th*^ and 75^*th*^ quantile frailty values, the hazard function depends on these values especially at the mid time (Figure 1). This frailty distribution introduced as an alternative to the gamma distribution [17] was better in this dataset compared to the gamma frailty distribution. The heterogeneity in the kebeles was estimated to be *θ* = 0.21, and the dependence within clusters is about *τ* = 8.1*%*. These values were the maximum among the variance of the random effects and the Kendall’s tau of all the models, which consolidates the idea that the better the model, the less unobserved heterogeneity will be [18].

Nonetheless, the most acknowledged parametric model is the weibull, which allows the proportional hazards and accelerated life time model [8]; the SAM data set was best described by the log-logistic baseline as compared to the exponential and weibull hazard functions. According to the diagnostic plots the *log failure odds* of log-logistic baseline with *log time* was more linear as compared to the plots of exponential (*cumulative hazard* versus *time*) and weibull (*log cumulative hazard* versus *log time*), showing the SAM dataset was best described by the log-logistic baseline. This result was also confirmed by the cumulative hazard plots for the Cox-Snell residuals of the exponential, weibull and the log-logistic models. The plot was more approached to the line in case of the log-logistic model, indicating that the log-logistic was best. A q-q plot was done to check if the accelerated failure time provided an adequate fit to the dataset and the log-logistic as baseline was accelerated failure time model. Hence, a survival model need not be chosen arbitrarily to fit event times, the baseline hazard function as well as the frailty distribution should be compared and the most appropriate model should be selected for appropriate inference.

The prognostic factors considered were the *age group* of the child, *type of malnutrition* and presence or absence of *co-infection*, which were significant covariates using univariable analysis. Analysis using the best model, log-logistic-inverse Gaussian frailty model showed that the *age group* of children and presence of *co-infection(s)* were the determinant factors for the time-to-cure from SAM. Children aged between 12–23 months had prolonged cure time as compared to older age groups. This *age group* is known to be the time when the prevalence of SAM is the highest [19–21]. This may be related to the fact that they start sub-optimal complementary feeding and compromise breast feeding practice which is important in preventing malnutrition among children [22]. Literatures like [23–25] identified infection as a prognostic indicators, likewise, co-infection(s) prolonged the time-to-cure from SAM in this study. However, our findings showed that the time of curing did not depend on *type of malnutrition*. Similarly, Efrem et al. [26] showed that there was no significant difference in average length of stay among patients with severe wasting and edematous malnutrition. Therefore, a special attention should be given to children with SAM between one to two years old and/or co-infected by other disease(s) in order to control excess numbers of children in the program at any one time which might increase the cost of the program. A program is well functioning and considered *acceptable* if the length of stay in a hospital is less than 4 weeks with acceptable cure proportion of greater than 75% [15]. This study revealed that the median time-to-cure from SAM for patients in Wolliso St. Luke Catholic hospital was 14 days with maximum cure time of 63 days of which (83.16%) were cured. This implies that the program was *acceptable* as per the above standard.

We classified SAM cases as marasmus, kwashiorkor and marasmic-kwashiorkor, a naming might imply that the cause is protein and calorie deficiency perse. However, the intent was to see if there are differential in recovery time between edematous and non-edematous cases of SAM. We acknowledge the limitation of our study for not being able to isolate SAM cases with micronutrient deficiency.

## Conclusion

The most appropriate statistical model for our dataset among various parametric frailty models, which well described the time-to-cure from SAM of the patients who were diagnosed in Wolisso St. Luke Catholic hospital is the log-logistic-inverse Gaussian frailty model. There is a frailty (clustering) effect on time-to-cure from SAM that arises due to heterogeneity between the kebeles of the children. The median curing time of the children is about 14 days with maximum cure time of 63 days of which 83.16% were cured. These values show *acceptable* functioning of the program in the hospital. Children who are aged between a year and two years and/or co-infected by other disease(s) prolonged their curing time as compared to the other groups of the patients among children under the age of five years.
